# The Role of the Intraplaque Vitamin D System in Atherogenesis

**DOI:** 10.1155/2013/620504

**Published:** 2013-12-26

**Authors:** Federico Carbone, Fabrizio Montecucco

**Affiliations:** ^1^Department of Internal Medicine, University of Genoa School of Medicine, IRCCS Azienda Ospedaliera Universitaria San Martino-IST Istituto Nazionale per la Ricerca sul Cancro, 6 Viale Benedetto XV, 16132 Genoa, Italy; ^2^Cardiology Division, Foundation for Medical Researches, Department of Internal Medicine, University of Geneva, 64, Avenue de la Roseraie, 1211 Geneva, Switzerland; ^3^Division of Laboratory Medicine, Department of Genetics and Laboratory Medicine, Geneva University Hospitals, 4 rue Gabrielle-Perret-Gentil, 1205 Geneva, Switzerland

## Abstract

Vitamin D has been shown to play critical activities in several physiological pathways not involving the calcium/phosphorus homeostasis. The ubiquitous distribution of the vitamin D receptor that is expressed in a variety of human and mouse tissues has strongly supported research on these “nonclassical” activities of vitamin D. On the other hand, the recent discovery of the expression also for vitamin D-related enzymes (such as 25-hydroxyvitamin D-1**α**-hydroxylase and the catabolic enzyme 1,25-dihydroxyvitamin D-24-hydroxylase) in several tissues suggested that the vitamin D system is more complex than previously shown and it may act within tissues through autocrine and paracrine pathways. This updated model of vitamin D axis within peripheral tissues has been particularly investigated in atherosclerotic pathophysiology. This review aims at updating the role of the local vitamin D within atherosclerotic plaques, providing an overview of both intracellular mechanisms and cell-to-cell interactions. In addition, clinical findings about the potential causal relationship between vitamin D deficiency and atherogenesis will be analysed and discussed.

## 1. Introduction

Since its discovery in the early 1900s, the role of vitamin D has been limited to calcium/phosphate homeostasis through a predominant action on the kidney, intestine, and bone [1]. On the contrary, evidence in recent decades has suggested that vitamin D might play a critical role in many other metabolic pathways, referred as “nonclassical effects” [2]. Thus, vitamin D is currently under investigation in cancer [3], autoimmune disorders [4], infections [5], and neurological [6] and cardiovascular (CV) diseases. A large amount of observational studies has shown that vitamin D deficiency is associated with a wide range of CV risk factors [7], as well as poor CV outcome [8], but more recent findings from interventional trial have weakened this initial enthusiasm with a more sceptical view. Ultimately, Brandenburg correctly stated: “there should be less persuasive observational associative data, but more convincing interventional results in the field of vitamin D” [9]. Certainly, a critical analysis of literature has revealed several limitations especially in study design, but also the newer insights about the local activity of vitamin D within peripheral tissues might explain the conflicting results between interventional and observational studies. In this new research approach, 25-hydroxyvitamin D-1*α*-hydroxylase (CYP27B1) is emerging as a main regulator of the extrarenal vitamin D system along with the catabolic enzyme 1,25-dihydroxyvitamin D-24-hydroxylase (CYP24A1) and vitamin D receptor (VDR). The aim of this review is to update the current evidence about the role of vitamin D in the pathophysiology of atherosclerosis and suggest a critical basis for future investigations.

## 2. Vitamin D Signalling

The availability of vitamin D is largely dependent on sunlight exposure (more than 80% of the requirements). In skin, ultraviolet-B (UVB) radiation induces the conversion of 7-dehydrocholesterol to the inactive precursor of vitamin D, through a photosynthetic reaction which evolved over 750 million years ago [10]. Subsequently, the 25-hydroxylation in the liver generates the 25-hydroxyvitamin D [25(OH) vitamin D or calcidiol] [11], which is biologically inactive but nonetheless used as marker of vitamin D status because of being stable, largely circulating, and easy to quantify. Calcidiol becomes active after conversion to 1,25-dihydroxyvitamin D [1,25(OH)_2_ vitamin D or calcitriol] which occurs through the action of CYP27B1, the rate-limiting enzyme [12]. Accordingly, CYP27B1 activity is tightly regulated with feedback control mechanisms (at least in the kidney) involving the parathyroid hormone (PTH), calcitonin, 1,25(OH)_2_ vitamin D itself [13], and CYP24A1 (the catabolic enzyme of vitamin D) [14]. The biological response to 1,25(OH)_2_ vitamin D is mediated by VDR, a DNA-binding transcription factor member of the nuclear receptor superfamily. VDR activation requires the binding to both 1,25(OH)_2_ vitamin D and one of retinoid X receptors (RXR *α*, *β*, or *γ*). Only in this heterodimeric form VDR complex recognizes the vitamin D response elements (VDRE), repeated sequences of 6 hexamers in the promoter region of target gene. Furthermore, since VDR may regulate 3% to 5% of human genome, allosteric influences, VDRE location, and epigenetic modification of DNA and histones modulate the VDR activity in the different cell types [15]. An additional feature shown by VDR (and by the whole nuclear receptor superfamily) is the ability to bind multiple lipophilic ligands, thus amplifying the vitamin D signalling activity. Interestingly, an extranuclear expression of VDR (on cell surface membrane and mitochondria) was recently discovered [16, 17] and shown to trigger nongenomic rapid responses [18].

Unlike the genomic responses (generally taking several hours till days to be fully manifest), these rapid nongenomic responses are generated in a shorter period of time (1-2 to 45 minutes). As already recognized for other steroidal hormones [19–21], plasma membrane caveolae are involved in vitamin D-induced rapid responses. Caveolae are localized within the lipid-rafts (microdomains of the plasma membrane enriched in sphingolipids and cholesterol) and might promote intracellular responses by flask-shaped membrane invagination [22]. VDR was found to be closely localized to caveolae [23], as also suggested by functional studies [24]. The VDR-caveolae complex may activate several downstream intracellular signalling cascades involving kinases, phosphatases, and ion channels as well as modulate gene expression, in a cross-talk with the classical genomic effects of vitamin D [25].

Ultimately, these recent insights, together with the ability to bind multiple lipophilic ligands (feature shared by the whole nuclear receptor superfamily), further increased complexity in vitamin D signalling pathways.

## 3. Vitamin D System and Atherosclerosis: Clinical Findings

Acute ischemic atherosclerotic complications are the leading cause of mortality and morbidity worldwide [26]. To date, it is commonly accepted that atherosclerotic plaque development is orchestrated by chronic low-grade inflammatory processes occurring within the arterial wall, in peripheral organs, and in the systemic circulation [27]. Endothelial dysfunction is a very early step in atherogenesis, especially at sites characterized by disturbed laminar flow. This pathophysiological event promotes subendothelial accumulation of low density lipoproteins (LDLs) [28]. Within the subintimal space of the arterial wall, LDLs (whether in native form or modified by oxidative stress) trigger inflammatory and vascular resident cells to produce several mediators attracting circulating leukocytes, including monocytes [29], neutrophils [30], and lymphocytes [31]. This chronic inflammatory process is responsible for the atherosclerotic plaque structure (including the necrotic lipid core and the fibrous cap) and promotes plaque instability [32]. Several observational studies and recent meta-analyses in humans showed that circulating 25(OH) vitamin D was inversely correlated with poor CV outcomes [8, 33, 34]. However, the first randomized clinical trials have provided even more discouraging results [34, 35]. In addition, also studies investigating the potential relationship between serum vitamin D and atherosclerotic plaque vulnerability have provided ambiguous results. For instance, studies focusing on carotid intima-media thickness (cIMT), a well-recognized biomarker of subclinical atherosclerosis also associated with a wide range of CV risk factors and CV diseases [36], showed a potential relationship between vitamin D deficiency and atherogenesis (Table 1). In particular, Deleskog and coworkers, in a longitudinal evaluation of 3,430 patients at high cardiovascular risk but without prevalent disease, failed to show an increased cIMT progression in vitamin D deficient patients when compared with the group with sufficient vitamin D [37]. On the other hand, the significant association between low vitamin D levels and a wide range of CV risk factor observed in this cohort did not prove any potential connections between vitamin D and clinical atherosclerotic outcomes. These recent findings are in accordance with previous observational studies. The research groups of Targher et al. and Liu et al. demonstrated an inverse correlation between vitamin D levels and cIMT severity [38, 39]. Among a subgroup of patients with end-stage renal disease, only Kraśniak and colleagues [40] showed a linear inverse correlation between 25(OH) vitamin D and cIMT. On the other hand, the case-control study of Briese et al. [41] and the cross-sectional analysis of Zang and coworkers [42] failed to prove any association. Likewise, in two observational cohorts of HIV-infected patients, vitamin D deficiency was showed as correlated with cIMT severity [43, 44]. However, these results were not confirmed by a recent larger simple size cross-sectional study [45]. Furthermore, recent studies (enrolling community-dwelling healthy subjects) failed to prove any relationships between vitamin D deficiency and cIMT. However, although these studies enrolled a large cohort of patients, they were designed with serious limitations. For instance, both geographical and seasonal differences in sunlight exposure might influence vitamin D status evaluation, as well as African race and old age. In addition, large simple size studies of vitamin D have been shown to underestimate other confounding factors, including differences in physical activity and dietary habits of patients, which may have significantly impacted the results [46–53]. As reported in Table 2, another CV surrogate parameter of atherogenesis (coronary artery calcium (CAC) score) has been used to investigate the potential direct association between low vitamin D levels and increased atherosclerotic plaque burden. An increased vascular calcification was previously associated with vitamin D deficiency in both experimental and clinical studies [54]. In particular, the most important clinical results were provided by the subgroup analyses from studies enrolling a large sample size. Mehrotra et al. reported a significant inverse correlation between vitamin D deficiency and CAC (prevalence and score) in patients with diabetic nephropathy [55]. On the other hand, both a recent study by Zang et al. [42] and a cross-sectional analysis from the Korean Longitudinal Study on Health and Aging did not confirm these results and failed to prove any relationships [51]. In accordance, additional cross-sectional analyses provided conflicting results [40, 46], whereas a predictive value of vitamin deficiency toward coronary calcification was supported by longitudinal studies such as the MESA (Multi-Ethnic Study of Atherosclerosis) (RR 1.38 (CI 95% 1.00–1.52); *P* = 0.04) [56] as well as a large cohort of patients with type I diabetes mellitus (RR 6.5 (CI 95% 1.1–40.2); *P* = 0.04) [57].

On the other hand, the stronger relationship between vitamin D deficiency and atherosclerosis has been demonstrated assessing endothelial dysfunction especially by flow-mediated dilatation (FMD) test (Table 3). Importantly, endothelial dysfunction is not only a predictor of future CV events [63] but also a very early marker of atherogenesis (also preceding angiographic or ultrasonic evidence of atherosclerotic plaque [64]). A large number of cross-sectional studies showed a significant and inverse correlation between vitamin D levels and ultrasound assessment of endothelial dysfunction (assessed by FMD test [60, 65–69] or measuring pulse wave velocity [62, 67, 70]), independently of other confounding parameters. In addition, the relationship between vitamin D deficiency and endothelial dysfunction was confirmed also investigating potential biochemical markers, such as interleukin (IL)-6 [65] and circulating endothelial progenitor cells [66]. Interestingly, a very recent study of Karohl and coworkers investigated the potential correlation between 25(OH) vitamin D and the coronary flow reserve (CFR) assessed by [(13)N]ammonia-positron emission tomography in asymptomatic middle-aged male twins. Low vitamin D levels were significantly correlated with CFR also in twin pairs, further supporting the role of vitamin D as a key player of endothelial function [71].

Unfortunately, although observational studies support a potential causal relationship between vitamin D deficiency and atherogenesis, randomized clinical trials have so far failed to demonstrate the beneficial effects of supplementation (Table 4). Although different treatment approaches supplementing vitamin D were shown as effective in increasing plasmatic 25(OH) vitamin D concentrations, their effects on CAC were ambiguous. However, these results were mainly provided by subgroup analyses of large randomized clinical trials that were not designed to assess this primary outcome [72, 73]. Similar results were provided by treatments targeting vitamin D supplementation on endothelial function. In fact, in several randomized clinical trials (with a similar sample size) showed that a short-term supplementation with vitamin D did not clearly improve endothelial dysfunction and virtually opposite results using different methods were found [74–80].

## 4. The Intraplaque Pathophysiological Activity of Vitamin D Axis

Recent studies suggest a local activity of vitamin D by an autocrine/paracrine mechanism. Evidence in support of this new paradigm includes the discovery of the expression of CYP27B1 (rate-limiting enzyme for vitamin D synthesis) as well as the VDR in several tissues and organs [81]. In this regard, Adams and Hewison have proposed that these newly discovered features of vitamin D biology are those phylogenetically more ancient, having been found also in single cell organisms and in species lacking calcified skeleton [82]. The first recognition of an extrarenal vitamin D system dates back more than twenty-five years ago, following studies of vitamin D metabolism in pregnancy [83] and granulomatous disease sarcoidosis [84]. Afterwards, some studies with knockout mice have demonstrated the expression of CYP27B1 in several other tissues, including skin [85], prostate [86], brain [87], pancreas [88], adipose tissue [89], skeletal muscle [90], heart [91], colon [92], and neoplastic tissues [93]. In 2012, Schnatz and coworkers firstly recognized the expression of VDR within atherosclerotic plaques of premenopausal cynomolgus monkeys [94], also observing an interesting inverse correlation between plaque burden and serum 25(OH) vitamin D levels [95]. Whether VDR expression might be suppressed by plaque progression or promote atherosclerotic vulnerability has not been clarified yet. However, these results might suggest that local activation of vitamin D could be involved in the pathophysiology of atherosclerosis although the recognition of VDR source has not been investigated yet.

### 4.1. Vitamin D Axis and Innate Immunity

Innate immunity, especially the mononuclear cell subset, is traditionally considered the main actor in atherosclerosis. The entire vitamin D system (including the hydroxylases CYP27A1 [96, 97] and CYP27B1 [98] as well as the VDR [99] and the vitamin D catabolic enzyme 24-hydroxylase [CYP24A1] [97]) was shown to be expressed in monocyte/macrophages. Starting from the observation that VDR deletion accelerated atherogenesis in LDL receptor knockout (LDLR^−/−^) mice, Szeto and coworkers observed that LDLR^−/−^/VDR^−/−^ bone marrow transplantation in LDLR^−/−^ recipients mice strongly promoted atherogenesis, thus pointing out the pivotal role of mononuclear cells as a main target for the protective administration of vitamin D against atherogenesis [100]. However, a significant breakthrough in this field was previously indicated by the observation that VDR-driven gene expression was upregulated in macrophages via the concomitant activation of toll-like receptor (TLR)4 [101–103], TLR1/2 [104], and TLR coreceptor CD14 [105]. In addition, VDR is a target gene for other intracellular pathways (such as those mediated by IL-15, which are involved in monocyte differentiation to macrophages [106]) and the T lymphocyte-released cytokines interferon (IFN)-*γ* [103] and IL-4 [107]. Interestingly, the local overexpression of 1,25(OH)_2_ vitamin D was shown to promote the inflammatory response enhancing the transcription of antimicrobial peptides (AMPs*β*-defensin 2 and cathelicidin) [108] and stimulating autophagy in atherosclerosis [109, 110] via a feedback mechanism [111]. On the other hand, vitamin D deficiency is associated with a pro-atherogenic monocyte phenotype (shift from M1 to M2 subtype) characterized by increased NF-*κ*B activity and TLR expression as well as enhanced endoplasmic reticulum stress and increased expression of adhesion molecules and proinflammatory cytokines [110, 112, 113]. Conversely, the activation of vitamin D signalling improves the macrophage response to lipid overload. Downregulating the expression of CD36 and the scavenger receptor (SR)-A1, 1,25(OH)_2_ vitamin D decreased the uptake of oxidized and acetylated LDLs and then the foam cell formation [114, 115]. In addition, 1,25(OH)_2_ vitamin D decreased cholesteryl ester formation and promoted a cholesterol efflux from macrophage in addition to suppressing their migration by downregulating the chemokine receptor CCR2 [116].

On the other hand, the role of neutrophils in atherogenesis and related disease has been unknown for long time, even because of being difficult to be recognized for of their short life-span and their plastic and dynamic properties [117]. Likewise, only recently, CYP27B1 has been discovered in neutrophils [118], whereas VDR expression was already detected [119]. Similar to mononuclear cells, an increased expression of VDR and CYP27B1 may act by a feedback mechanism on activated neutrophils, decreasing the synthesis of proinflammatory molecules, such as CXCL8 [119], macrophage inflammatory protein (MIP)-1*β*, IL-1*β*, and vascular endothelial growth factor.

### 4.2. Vitamin D Axis and Adaptive Immunity

Through their role of antigen presenting cells, dendritic cells (DCs) are essential for both innate and adaptive immune systems functioning [120]. DCs have been largely recognized in the wall of healthy arteries, but their role in atherogenesis still remains unclear [121]. As reported by Gautier and colleagues, the transplantation of apoptosis-resistant DCs in LDLR^−/−^ recipients mice failed to accelerate plaque progression, despite the fact that this experimental model exhibited a proatherogenic pattern characterized by increased T-cell activation (with a shift toward the proatherogenic Th1 phenotype) and a rise in circulating levels of antibodies against oxidized LDL (oxLDL) [122]. Conversely, a reduced atherosclerotic burden was directly correlated with a reduced DC recruitment (as observed in mice lacking CXC3R1, CCL2, and CCR5 [123–125]). DCs are a major source of vitamin D since they constitutively express high level of CYP27B1, that are enhanced after TLR stimulation (both TLR4 [126] and TLR1/2 [106, 127]). Through an autocrine loop, 1,25(OH)_2_ vitamin D was shown to suppress DC differentiation/activation up to induce a regression of differentiated DCs toward a more immature stage [128]. Additional effects of 1,25(OH)_2_ vitamin D on DCs include impairment on cell chemotaxis [128] and suppression of proinflammatory cytokines (e.g., IL-1 and tumor necrosis factor-*α*). In addition, 1,25(OH)_2_ vitamin D might promote a more tolerogenic phenotype of DCs decreasing the expression of class 2 MHC molecules, CD40, CD80, and CD86 [129, 130].

On the other hand, the regression of atherosclerotic burden induced by 1,25(OH)_2_ vitamin D might occur also via a direct effect on T cells [131–133] and this is consistent with several lines of evidence supporting atherosclerosis as a T-cell-driven disease [134]. Targeting more than 102 genes in CD4^+^ T cells, 1,25(OH)_2_ vitamin D-VDR signalling might importantly regulate T-cell activity, especially the T-helper (Th) polarization, skewing from the proinflammatory phenotype Th1 and Th17 (by suppressing IFN-*γ*, IL-2, and IL-17) towards an anti-inflammatory Th2 phenotype (by promoting IL-4 and IL-5 gene transcription) [135]. In addition, a recent study by Yadav and colleagues has demonstrated an association between vitamin D deficiency to an increased CD4^+^ CD28^+^ T-lymphocyte count [136] (a proatherogenic T-cell subtype) [137].

Interestingly, following the discovery that FOXP3 transcription is directly targeted by VDR [138], also some beneficial effects of vitamin D might involve the regulatory T-cell (Treg) subtype [139–141] that has been described to reduce atherosclerosis [131–133].

Overall, the immunomodulation exerted by locally activated vitamin D system on the adaptive immune system relies not only on an autocrine loop (in addition to DCs, CYP27B1 has been recognized also in T cells [142]) but especially on a paracrine effect regulated by a complex cross-talks between different cell types (for instance the combined stimulation with CD40/CD40 ligand and cytokines is the strongest inducer of CYP27B1 synthesis in DCs [143]).

### 4.3. The Potential Interactions between Vitamin D and Other Endocrine Pathways

Molecular and cellular mechanisms of atherogenesis and atheroprogression were shown to involve the upregulation of several neurohormonal mediators. One of the best-known hormonal axes is the renin angiotensin aldosterone system (RAAS). In particular through angiotensin II, RAAS was shown to increase vascular injury by enhancing the oxidative stress-mediated pathways and systemic inflammatory responses [144]. Moreover, a local vascular activity of RAAS has been also suggested by the detection of the expression of the angiotensin converting enzyme within atherosclerotic lesions [145]. Vitamin D is a well-known negative regulator of RAAS [146, 147] and this feature is emerging as potential pathway potentially involved in vascular injury prevention. By deleting VDR gene in LDLr^−^/^−^ mice, Szeto and coworkers firstly suggested that inhibition of macrophage VDR signalling in atherosclerotic mice also suppressed the RAAS [100]. Further studies by Ish-Shalom and colleagues and Weng and coworkers have recently supported these findings in mice [148, 149]. In addition, the discovery of fibroblast growth factor (FGF)23/klotho axis has also broadened the potential role of vitamin D on the endocrine signalling in the pathogenesis of atherosclerosis. FGF23 was shown to act as counterregulatory hormone of vitamin D, suppressing both renal and extrarenal synthesis of CYP27B1 as well as enhancing the expression of catabolic enzyme CYP24A1. FGF23 is also a well-recognized risk factor for CV diseases and CV mortality [150, 151]. In addition, evidence of its direct role in promoting atherosclerosis also in patients with preserved renal function was also demonstrated [152]. Although the molecular mechanisms underlying both FGF23 and vitamin D still require to be clarified [153], recent pathophysiological studies have shown potential biphasic cardiovascular effects of these mediators in atherogenesis associated with chronic renal diseases [154].

## 5. Conclusions

In the last decades, the scientific debate on the CV effects of vitamin D system and the potential CV risk associated with its deficiency raised controversial findings [155]. Even if the results from the first randomized clinical trials were discouraging, these studies were not considered conclusive at all, due to limitations in study design and different compounds administered. Poor stratification by age, race, geographic position, physical activity, and sunlight exposure were the main confounding factors, in addition to the small sample size of cohorts. Moreover, the current definitions of the optimal vitamin D level in humans are bone-driven and not assessed from a cardiovascular point of view. In addition, the different compounds used for vitamin D supplementation (comprising both inactive forms of vitamin D and direct VDR agonists) may affect the reliability of these results.

On the other hand, the contribution of the local activated vitamin D system within atherosclerotic plaque has not been appropriately investigated yet. Therefore, both basic research studies and clinical trials are needed for better elucidating the therapeutic and pathophysiological role of vitamin D in atherogenesis and CV diseases.

## Figures and Tables

**Table 1:** Observational studies investigating the relationship between vitamin D and carotid intima-media thickness.

Author	Year	Study design (sample size)	Country (ethnicity) Age	Correlation (lower range of 25(OH)D)	Findings
Briese et al. [41]	2006	Case-control (40 ESRD patients and 40 matched healthy controls)	Germany (Caucasian) Mean 23.6 years	No (linear correlation)	There was no difference in CCA-IMT between the two groups. This study failed to correlate 25(OH)D and cIMT.
Targher et al. [38]	2006	Case-control (390 TDM2 patients and 390 healthy controls)	Italy (Caucasian) 50–65 years	Yes (<37.5 nmol/L)	Low 25(OH)D level independently predicted CCA-IMT (*P* < 0.001).
Kraśniak et al. [40]	2007	Cross-sectional (73 patients in haemodialysis)	Poland (Caucasian) 25–75 years	Yes (linear correlation)	The study showed a linear inverse correlation between 25(OH)D and CCA-IMT at univariate analysis (*P* < 0.01).
Michos et al. [46]	2009	Cross-sectional (650 Amish people)	U.S. (Caucasian) Stratified	No (I quartile < 18.1 nmol/L)	The study failed to detect an association between 25(OH)D and cIMT.
Pilz et al. [47]	2009	Prospective observational (614 subjects from the Hoorn study)	Netherlands (Caucasian) Mean 68.5 years	No (not provided)	This post hoc analysis failed to detect an association between 25(OH)D and cIMT.
Reis et al. [48]	2009	Cross-sectional (654 older adults from Rancho Bernardo Study)	U.S. (Caucasian) 55–96 years	Yes (I quartile < 32.0 nmol/L)	In this study, 25(OH)D was associated with geometric mean internal cIMT (*P* for trend = 0.02) but not CCA-IMT. Instead 1,25(OH)_2_D or PTH did not correlate with IMT.
Hajas et al. [58]	2011	Cross-sectional (125 females MCTD patients)	Hungary (Caucasian) Mean 53.6	Yes (linear correlation)	The study reported a significant linear inverse association between 25(OH)D and cIMT (*P* < 0.001).
Richart et al. [49]	2011	Cross-sectional (542 females from FLEMENGHO study)	Belgium (Caucasian) Stratified	No (linear correlation)	cIMT was associated with PTH/25(OH)D ratio (*P* < 0.01), not with 25(OH)D alone.
Choi et al. [43]	2011	Cross-sectional (139 HIV-infected subjects)	U.S. (Caucasian and African Americans) Mean 45 years	Yes (<37.5 nmol/L)	At adjusted analysis, 25(OH)D insufficiency was associated with higher mean cIMT levels (*P* = 0.02).
Ross et al. [44]	2011	Case-control (149 HIV-infected subjects)	U.S. (Caucasian and African Americans) Not provided	Yes (not provided)	At adjusted analysis, 25(OH)D insufficiency increased risk of CCA-IMT (OR 10.62 (CI 95% 1.37–82.34); *P* < 0.01).
Pacifico et al. [59]	2011	Cross-sectional (452 children and adolescent)	Italy (Caucasian) Stratified	No (I tertile < 42.5 nmol/L)	This post hoc analysis failed to detect an association between 25(OH)D and cIMT.
Carrelli et al. [50]	2011	Cross-sectional (203 subjects from the Northern Manhattan study)	U.S. (Caucasian and African Americans) 50–93 years	Yes (linear correlation)	Multiple regression analysis showed an independent inverse correlation of 25(OH)D with cIMT (*P* = 0.05) as well as total plaque thickness (*P* = 0.03).
Shikuma et al. [45]	2012	Cross-sectional (1003 HIV-infected subjects from the HAHC-CVD)	U.S. (Caucasian and other races) Mean 52 years	No (I tertile < 25 nmol/L)	This cohort did not show any correlation between 25(OH)D and cIMT.
Lim et al. [51]	2012	Cross-sectional (921 subjects from the KLoSHA)	Korea (Asian) Mean 76	No (<37.5 nmol/L)	This study failed to prove a correlation between 25(OH)D and cIMT.
Liu et al. [39]	2012	Cross-sectional (300 TDM2 patients)	China (Asian) Not provided	Yes (<26.17 nmol/L)	Lower 25(OH)D levels inversely correlated with cIMT (*P* for trend < 0.05) also at multivariate analysis (*P* < 0.01). Similar findings were observed comparing patients with and without carotid atherosclerosis (*P* < 0.01).
Knox et al. [52]	2012	Cross-sectional (625 healthy subjects from pSoBid study)	UK (not provided) 35–64	No (linear correlation)	There was no evidence of an association of increasing 25(OH)D with risk of plaque presence or cIMT in the whole group in univariate or adjusted models.
Zang et al. [42]	2012	Cross-sectional (151 patients with diabetic nephropathy)	China (Asian) Not provided	No (<37.5 nmol/L)	This study failed to prove a correlation between 25(OH)D and cIMT.
Oz et al. [60]	2013	Cross-sectional (222 patients undergoing coronary angiography)	Turkey (Turkish) Stratified	Yes (<75 nmol/L)	The vitamin D deficient group showed an independent and inverse correlation with cIMT (*P* < 0.001)
Blondon et al. [53]	2013	Cross-sectional and longitudinal (3251 subjects from the Multi-Ethnic Study of Atherosclerosis)	US (Caucasian, African Americans, Asian, Hispanic) Mean 60 years	No (<50 nmol/L)	At multivariate analysis 25(OH)D failed to correlate with cIMT both in cross-sectional and in longitudinal analysis.
Kiani et al. [61]	2013	Longitudinal observational (154 patients from the LAPS)	US (Caucasian, African Americans, and others) Stratified Mean 46 years	No (<52.5 nmol/L)	After 2 years of follow-up, this study failed to prove a correlation between 25(OH)D and cIMT.
Sypniewska et al. [62]	2014	Cross-sectional (98 hypertensive patients)	Poland (Caucasian) 42–58 years	Yes (<52.5 nmol/L)	In this cohort, 25(OH)D was inversely correlated with cIMT (*P* < 0.02).
Deleskog et al. [37]	2013	Longitudinal observational (3430 subjects with high CV risk)	Europe (not provided) Mean 64 years	No (<25 nmol/L)	25(OH)D correlated with CV risk factors but not with cIMT progression after 30 months follow-up.

ESRD: end-stage renal disease; CCA-IMT: common carotid artery intima-media thickness; cIMT: carotid intima-media thickness; TDM2: type 2 diabetes mellitus; PTH: parathyroid hormone; MCTD: mixed connective tissue disease; FLEMENGHO: Flemish Study on Environment, Genes and Health Outcomes; HIV: human immunodeficiency virus; OR: odds ratio; CI: confidence of interval; HAHC-CVD: Hawaii aging with HIV-cardiovascular; KLoSHA: Korean Longitudinal Study on Health and Aging; pSoBid: psychological, social and biological determinants of ill health; LAPS: Lupus Atherosclerosis Prevention Study; and CV: cardiovascular.

**Table 2:** Observational studies investigating the relationship between vitamin D and carotid artery calcification.

Author	Year	Study design (sample size)	Country (ethnicity) Age	Correlation (lower range of 25(OH)D)	Findings
Kraśniak et al. [40]	2007	Cross-sectional (73 patients in haemodialysis)	Poland (Caucasian) 25–75 years	Yes (linear correlation)	The study showed a linear correlation between 25(OH)D and CAC (*P* < 0.01).
Mehrotra et al. [55]	2008	Cross-sectional (146 patients with diabetic nephropathy form NHANES III)	U.S. (Caucasian, African Americans, and Hispanic) Stratified	Yes (<37.5 nmol/L)	In 25(OH)D deficient group, there was higher CAC prevalence (*P* = 0.002) and score (*P* = 0.04).
Michos et al. [46]	2009	Cross-sectional (650 Amish people)	U.S. (Caucasian) Stratified	No (I quartile < 75 nmol/L)	The study failed to detect an association between 25(OH)D and CAC.
De Boer et al. [56]	2009	Longitudinal observational (1370 subjects from MESA)	U.S. (Caucasian, African Americans, and Hispanic) Stratified	Yes (<37.5 nmol/L)	After 3 years of follow-up, vitamin D deficient group had increased incidence of CAC at multivariate analysis (RR 1.38 (CI 95% 1.00–1.52); *P* = 0.04).
Young et al. [57]	2011	Longitudinal observational (374 TDM1 patients)	U.S. (Caucasian) Mean 39 years	Yes (<50 nmol/L)	After 3 years of follow-up, 25(OH)D deficiency was associated with higher incidence of CAC at multivariate analysis (RR 6.5 (CI 95% 1.1–40.2); *P* = 0.04).
Shikuma et al. [45]	2012	Cross-sectional (1003 HIV-infected subjects from the HAHC-CVD)	U.S. (Caucasian and other races) Mean 52 years	Yes (I tertile < 25 nmol/L)	Lower 25(OH)D was associated with slightly higher risk of having CAC (RR 1.02; *P* = 0.04) without a linear correlation between 25(OH)D and CAC score.
Lim et al. [51]	2012	Cross-sectional (921 subjects from the KLoSHA)	Korea (Asian) Mean 76	No (<37.5 nmol/L)	This study failed to prove a correlation between 25(OH)D and CAC score.
Zang et al. [42]	2012	Cross-sectional (151 patients with diabetic nephropathy)	China (Asian) Not provided	No (<37.5 nmol/L)	CAC was not correlated with 25(OH)D but only with 1,25(OH)_2_D3 (*P* < 0.05).

CAC: coronary artery calcification; NHANES III: Third National Health and Nutrition Examination Survey; MESA: Multi-Ethnic Study of Atherosclerosis; RR: relative risk; CI: confidence interval; TDM1: type 1 diabetes mellitus; HIV: human immunodeficiency virus; HAHC-CVD: Hawaii Aging with HIV-Cardiovascular; and KLoSHA: Korean Longitudinal Study on Health and Aging.

**Table 3:** Observational studies investigating the relationship between vitamin D and endothelial dysfunction.

Author	Year	Study design (sample size)	Country (ethnicity) Age	Correlation (lower range of 25(OH)D)	Findings
Jablonski et al. [65]	2011	Cross-sectional (75 subjects)	U.S. (Caucasian, Hispanic and Asian) 50–79 years	Yes (<50 nmol/L)	Brachial FMD was lower in vitamin D-deficient group (*P* < 0.01), showing a linear correlation with 25(OH)D (*P* < 0.01). Moreover, 25(OH)D showed a significant inverse correlation with IL-6 (*P* < 0.01) and CYP27B1 (*P* < 0.05).
Yiu et al. [66]	2011	Cross-sectional (280 TDM2 and 73 matched healthy subjects)	Hong Kong (not provided) Stratified	Yes (<50 nmol/L)	Vitamin D-deficient group showed lower brachial FMD (*P* = 0.003). In addition, there was a significant linear correlation between low 25(OH)D levels and CD133+/KDR+ EPC (*P* < 0.001).
Al Mheid et al. [67]	2011	Cross-sectional (554 healthy subjects)	U.S. (Caucasian, African Americans and Hispanic) Mean 47 years	Yes (linear correlation)	25(OH)D was independently correlated with brachial FMD (*P* = 0.03) and PWV (*P* = 0.04).
Chitalia et al. [68]	2012	Cross-sectional (50 CKD patients)	U.K. (not provided) 15–85 years	Yes (linear correlation)	This study showed a linear correlation between 25(OH)D and brachial FMD (*P* = 0.007).
Syal et al. [69]	2012	Cross-sectional (100 patients undergoing coronary angiography)	India (Indian) Mean 56 years	Yes (<50 nmol/L)	25(OH)D was independently correlated with brachial FMD (*P* = 0.002).
Karohl et al. [71]	2013	Cross-sectional (368 soldiers from the Vietnam Era Registry)	U.S. (Caucasian 93.5%) Mean 55 years	Yes (<75 nmol/L)	CFR assessed with PET [^13^N]ammonia was lower in vitamin D-deficiency group (*P* = 0.007).
Oz et al. [60]	2013	Cross-sectional (222 patients undergoing coronary angiography)	Turkey (Turkish) Stratified	Yes (<75 nmol/L)	Patients with vitamin D deficiency has slower coronary flow (RR 3.5 (CI 95% 1.1–10.5); *P* = 0.01). In addition, 25(OH)D deficiency correlated independently with FMD (*P* < 0.001)
Kuloglu et al. [70]	2013	Cross-sectional (133 hypertensive patients)	Turkey (Caucasian) Mean 62 years	Yes (not available)	In this cohort 25(OH)D showed a signficant correlation with PWV (*r* = −0.432; *P* < 0.001)
Sypniewska et al. [62]	2014	Cross-sectional (98 hypertensive patients)	Poland (Caucasian) 42–58 years	Yes (<52.5 nmol/L)	In this cohort 25(OH)D showed a signficant correlation with PWV (*r* = −0.33; *P* = 0.03)

FMD: flow-mediated dilation; IL: interleukin; CYP27B1: 25-hydroxyvitamin D-1-*α* hydroxylase; TDM2: type 2 diabetes mellitus; PWV: pulse wave velocity; CKD: chronic kidney disease; CFR: coronary flow reserve.

**Table 4:** Interventional studies investigating the relationship between vitamin D deficiency and atherosclerosis.

Author	Year	Study design (sample size)	Country (ethnicity) Age	Intervention (follow-up)	Findings
Coronary artery calcification					
Manson et al. [72]	2010	Prospective randomized double-blind placebo-controlled trial (750 postmenopausal women from the WHI-CACS)	U.S. (Caucasian, Africa Americans, Hispanic, and Asian) 50–59 years	Calcium 500 mg ×2/day or calcium 500 mg + 25(OH)D 5 *μ*g twice daily or placebo (7 years)	After follow-up, CAC measurements were similar in both groups also at multivariate analysis.
Raggi et al. [73]	2011	Prospective randomized double-blind controlled trial (360 patients in haemodialysis from the ADVANCE trial)	U.S. (Caucasian, Africa Americans, Hispanic, and Asian) Mean 61 years	Cinecalcet (30–180 mg/day) + active vitamin D or vitamin D alone (20 weeks for titration and after 32 weeks of follow-up)	After 52 weeks, treatment with cinecalcet significantly slowed vascular calcification (*P* = 0.009)

Endothelial dysfunction					
Sudgen et al. [74]	2008	Prospective randomized double-blind controlled trial (34 TDM2 patients)	U.K. (not provided) Mean 64 years	Ergocalciferol loading dose 100.000 U or placebo (8 weeks)	Vitamin D supplementation improves FMD (*P* = 0.04), in addition to reducing systolic BP (*P* = 0.001) and increasing serum levels of 25(OH)D (*P* = 0.02)
Tarcin et al. [75]	2009	Longitudinal interventional (23 subjects with 25(OH)D < 75 nmol/L)	Turkey (not provided) Stratified	Ergocalciferol loading dose 300.000 U/monthly ×3 doses (3 months)	Treatment significantly improved FMD (*P* = 0.002)
Witham et al. [76]	2010	Prospective randomized double-blind placebo-controlled trial (61 TDM2 patients)	U.K. (not provided) Stratified	Ergocalciferol loading dose 100.000 U or 200.000 U or placebo (8 and 16 weeks)	Supplementation significantly raised serum 25(OH)D levels but failed to improve FMD.
Shab-Bidar et al. [77]	2011	Prospective randomized double-blind controlled trial (100 TDM2 patients)	Iran (not provided) 29–67 years	Fortified diet with Ca^++^ 170 mg/or Ca^++^ 170 mg + 25(OH)D 12.5 *μ*g twice a day (12 weeks)	Supplementation improved endothelial function evaluated through adjusted endothelin-1 (*P* = 0.009) and MMP-9 (*P* = 0.005) assay.
Witham et al. [78]	2012	Prospective randomized double-blind placebo-controlled trial (34 TDM2 patients)	U.K. (not provided) Stratified	Ergocalciferol loading dose 100.000 or placebo (8 and 16 weeks)	Supplementation significantly improves FMD at 8 weeks (*P* = 0.007) but not at 16 weeks.
Stricker et al. [79]	2012	Prospective randomized double-blind placebo-controlled trial (76 patients with PAD)	Swiss (Caucasian) Stratified	Ergocalciferol loading dose 100.000 or placebo (1 months)	Supplementation significantly raised serum 25(OH)D levels but failed to improve arterial stiffness.
Yiu et al. [80]	2013	Prospective randomized double-blind placebo-controlled trial (100 TDM2 patients with 25(OH)D < 75 nmol/L)	Hong Kong (not provided) Mean 65 years	25(OH)D 125 *μ*g/day or placebo (12 weeks)	Supplementation significantly raised serum 25(OH)D and Ca^++^ concentration in addition to decreasing PTH. However, the study failed to improve vascular function assessed by FMD circulating EPCs and PWV.

WHI-CACS: Women's Health Initiative Coronary Artery Calcium Study; CAC: coronary artery calcification; ADVANCE: Study to Evaluate Cinacalcet Plus Low Dose Vitamin D on Vascular Calcification in Subjects With Chronic Kidney Disease Receiving Hemodialysis; TDM2: type 2 diabetes mellitus; FMD: flow mediated dilatation; PAD: peripheral artery disease; EPCs: endothelial progenitor cells; and PWV: pulse wave velocity.
